# The safety profile of Bald’s eyesalve for the treatment of bacterial infections

**DOI:** 10.1038/s41598-020-74242-2

**Published:** 2020-10-15

**Authors:** Blessing O. Anonye, Valentine Nweke, Jessica Furner-Pardoe, Rebecca Gabrilska, Afshan Rafiq, Faith Ukachukwu, Julie Bruce, Christina Lee, Meera Unnikrishnan, Kendra P. Rumbaugh, Lori A. S. Snyder, Freya Harrison

**Affiliations:** 1 School of Life Sciences, University of Warwick, Coventry, UK; 2 School of Life Sciences, Pharmacy, and Chemistry, Kingston University, Kingston upon Thames, UK; 3 Department of Surgery, Texas Tech University Health Sciences Center School of Medicine, Texas, USA; 4 Warwick Clinical Trials Unit, Warwick Medical School, University of Warwick, Coventry, UK; 5 School of English and Centre for the Study of the Viking Age, University of Nottingham, Nottingham, UK; 6 Microbiology and Infection Unit, Warwick Medical School, University of Warwick, Coventry, UK; 7 Warwick Medical School, University of Warwick, Coventry, UK; 8 Present Address: School of Medicine, University of Central Lancashire, Preston, PR1 2HE UK

**Keywords:** Drug safety, Toxicology, Microbiology

## Abstract

The rise in antimicrobial resistance has prompted the development of alternatives to combat bacterial infections. Bald’s eyesalve, a remedy used in the Early Medieval period, has previously been shown to have efficacy against *Staphylococcus aureus *in in vitro and in vivo models of chronic wounds. However, the safety profile of Bald’s eyesalve has not yet been demonstrated, and this is vital before testing in humans. Here, we determined the safety potential of Bald’s eyesalve using in vitro, ex vivo, and in vivo models representative of skin or eye infections. We also confirmed that Bald’s eyesalve is active against an important eye pathogen, *Neisseria gonorrhoeae.* Low levels of cytotoxicity were observed in eyesalve-treated cell lines representative of skin and immune cells. Results from a bovine corneal opacity and permeability test demonstrated slight irritation to the cornea that resolved within 10 min. The slug mucosal irritation assay revealed that a low level of mucus was secreted by slugs indicating moderate mucosal irritation. We obtained promising results from mouse wound closure experiments; no visible signs of irritation or inflammation were observed. Our results suggest that Bald’s eyesalve could be tested further on human volunteers to assess safety for topical application against bacterial infections.

## Introduction

There is an urgent need for the development of new antimicrobials to fight bacterial infections which have become resistant to antibiotics. Antimicrobial resistance (AMR) now poses a serious threat to human health, especially to those with a suppressed immune system. AMR has an associated mortality of over 33,000 people per annum in Europe, with a prediction that 10 million people per year are likely to be killed by 2050 globally^1–4^. This rise in AMR, coupled with the lack of development of new antibiotics, has prompted research into the use of alternatives, such as plant-derived compounds^5–7^.

Historically, plant-based compounds have been explored for their antimicrobial properties. These include oils from *Cupressus sempervirens *(Cypress) and *Commiphora* species (myrrh), being used to treat colds, coughs, and inflammation^8^, and *Artemisia annua* (Artemisin) used to treat malaria^9^. Bald’s eyesalve has previously been shown to possess antibacterial activity against *Staphylococcus aureus* in planktonic cultures and biofilms^10^, and against *Pseudomonas aeruginosa* in planktonic cultures^11^. A recent study from our laboratory found that Bald’s eyesalve had antimicrobial activity against a range of Gram-positive and Gram-negative organisms in planktonic cultures and biofilms^12^. Furner-Pardoe et al., also demonstrated that all the ingredients specified in the original translation of Bald’s eyesalve were necessary for its anti-biofilm activity^12^. However, the safety profile of the eyesalve has not previously been reported. To explore whether this eyesalve could potentially be effective against bacterial infections in humans, it is important to firstly determine the safety profile.

Traditionally, animals have been employed for safety testing of compounds, but in order to reduce the number of animals used in line with the 3Rs (replacement, reduction and refinement of animal use)^13^, we decided to use alternate models. Cell lines are particularly robust for measuring the cytotoxic effects of drug formulation and appropriate cell lines been used as surrogates for different body sites. Human cell lines, HaCaT and THP-1, have been used as substitutes for human skin cells and immune cells respectively. These cell lines have been employed for cytotoxicity testing of other natural products, drugs, and cosmetic products^13–16^.

The bovine corneal opacity and permeability (BCOP) test is an eye irritation assay that replaced the Draize rabbit test (an in vivo assay) which identifies chemicals/irritants that induce serious eye damage^17,18^. It is described in Sect. 4 of the Organisation for Economic Co-operation and Development’s guidelines (Test No 437, https://doi.org/10.1787/9789264203846-en) for assays that can be used to determine the health effects of chemicals. The BCOP assay assesses the effect of potential irritants on the opacity and permeability of the isolated corneas. Toxicity of the test substance is measured by decreased light transmission leading to opacity, indicating corneal injury, and increased fluorescein dye permeability, reflecting damage to the corneal epithelium. This assay acts as an alternative to the use of animals in initial toxicity testing as the isolated bovine eyes are abattoir by-products that would otherwise be disposed of. Churchward and colleagues used the BCOP test to determine that antimicrobial fatty acids proposed for use in prophylaxis and treatment of gonococcal eye infections do not cause irritation^19^. It was also employed to test tacrolimus, an immunosuppressive drug for the treatment of autoimmune-based inflammatory eye disorders^20^.

Similarly, the slug mucosal irritation (SMI) assay is an alternative to the Draize test that has been used in testing the eye irritation potency of ingredients in cosmetic products, such as shampoos^21^. The premise of the SMI test is that mucus will be produced when slugs are exposed to irritating substances and the slugs will lose weight. Hence, irritants will cause weight loss, mucus production, and release of proteins and enzymes due to tissue damage^22^. This can be indirectly correlated to the effects of substances on humans, such as the stinging, itching and burning normally experienced when eyes are irritated, leading to red and watery eyes^21^.

The aim of this present study was to determine the safety and irritation potential of Bald’s eyesalve using alternative in vitro and ex vivo models to consider safety parameters before undertaking testing on humans. Human cell lines were employed as an initial screen, followed by the BCOP test and slug mucosal irritation assays. Given the original prescription of the remedy was to treat eye infections, we also confirmed the activity of the eyesalve against *Neisseria gonorrhoeae*, a common cause of neonatal conjunctivitis. Finally, due to the low levels of cytotoxicity observed in our in vitro and ex vivo assays, we performed wound healing experiments in mouse models. This was necessary as our research is primarily geared towards the topical application of the eyesalve, or application of the active ingredients therein, in the treatment of bacterial infections such as those affecting the skin and eyes.

## Results

### Bald’s eyesalve is active against *Neisseria gonorrhoeae*

**Figure 1:**
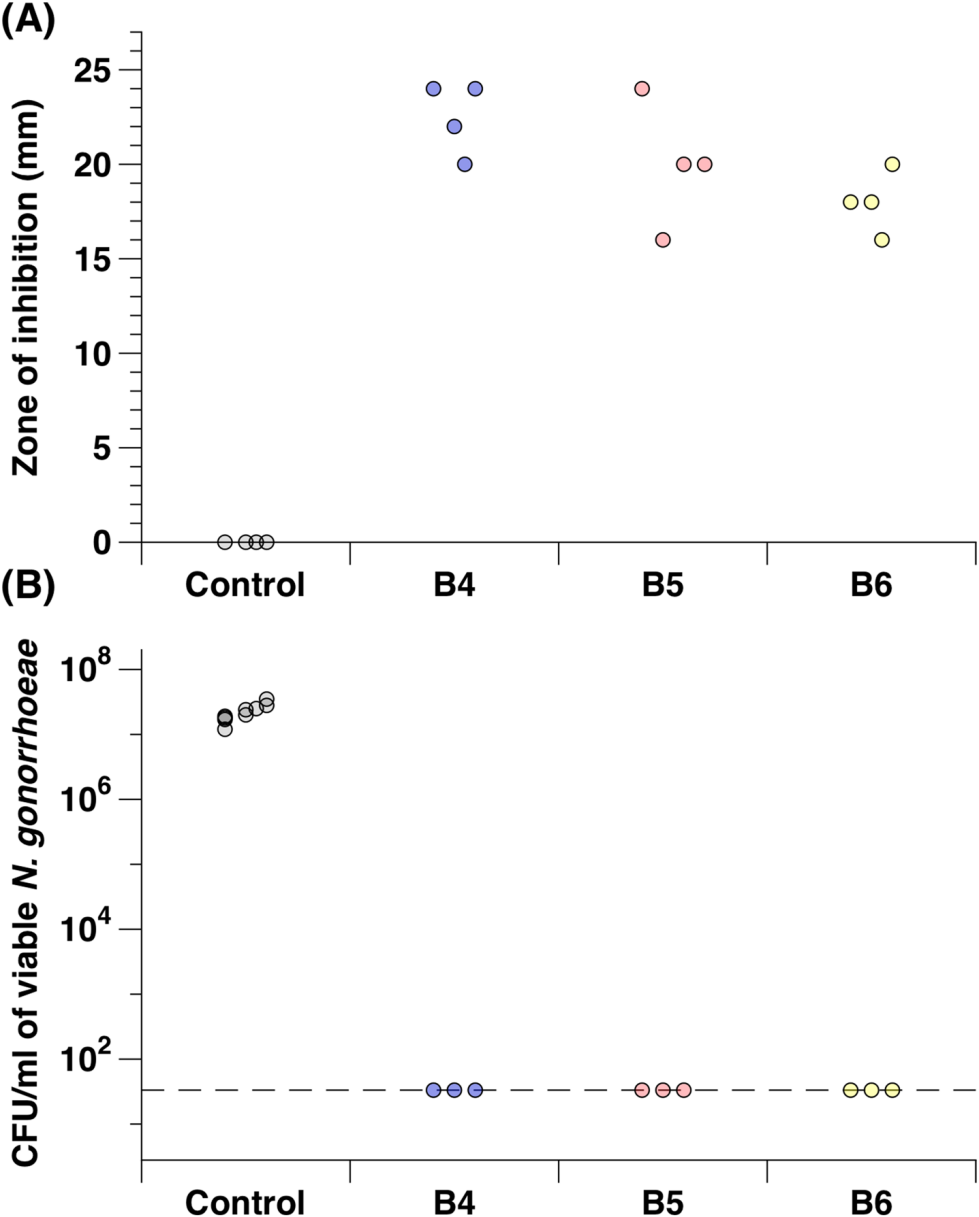
Antibacterial activity of Bald’s eyesalve against *N. gonorrhoeae*. (**A**) Zones of inhibition of three batches of Bald’s eyesalve (B4, B5 and B6) against *N. gonorrhoeae* NCCP11945.The control used was sterile distilled water and n = 4 replicates per treatment including the control. We found differences between the control and the three eyesalve batches (Kruskal–Wallis test on the full data set H_3_ = 11.7, *p* = 0.008; dropping the control treatments H_2_ = 5.16, *p* = 0.076). (**B**) The number of colonies obtained after treatment with three batches of eyesalve. The control used was PBS and n = 3 replicates per treatment and 9 replicates for the control as separate controls was run for the three different eyesalve batches. The dashed line represents the limit of detection.

We have previously shown that Bald’s eyesalve is active against *S. aureus* in planktonic culture, in biofilms formed in an artificial model of wound infection, and in biopsies taken from infected mouse wounds^10^. Here, we wanted to investigate a further eye pathogen of clinical interest. We choose *N. gonorrhoeae* as it is a pathogen that causes neonatal conjunctivitis. We performed disk diffusion assays using sterile 6 mm disks infused with 10 µl of three batches of eyesalve placed on a gonococcal agar plate containing the multi-drug resistant *N. gonorrhoeae* NCCP11945 strain. The results showed that Bald’s eyesalve was active against this strain as significantly larger zones of inhibition were obtained compared to the control, and zone size did not significantly differ between batches (Fig. 1a, Kruskal–Wallis test on the full data set H_3_ = 11.7, *p* = 0.008; dropping the control treatments H_2_ = 5.16, *p* = 0.076). Furthermore, planktonic cultures of this strain were exposed briefly to the three batches of the eyesalve and a 7-log reduction of *N. gonorrhoeae* was observed compared to the control, as no viable colonies were found on the eyesalve-treated cultures (Fig. 1b).

### The viability of human cells treated with Bald’s eyesalve

**Figure 2:**
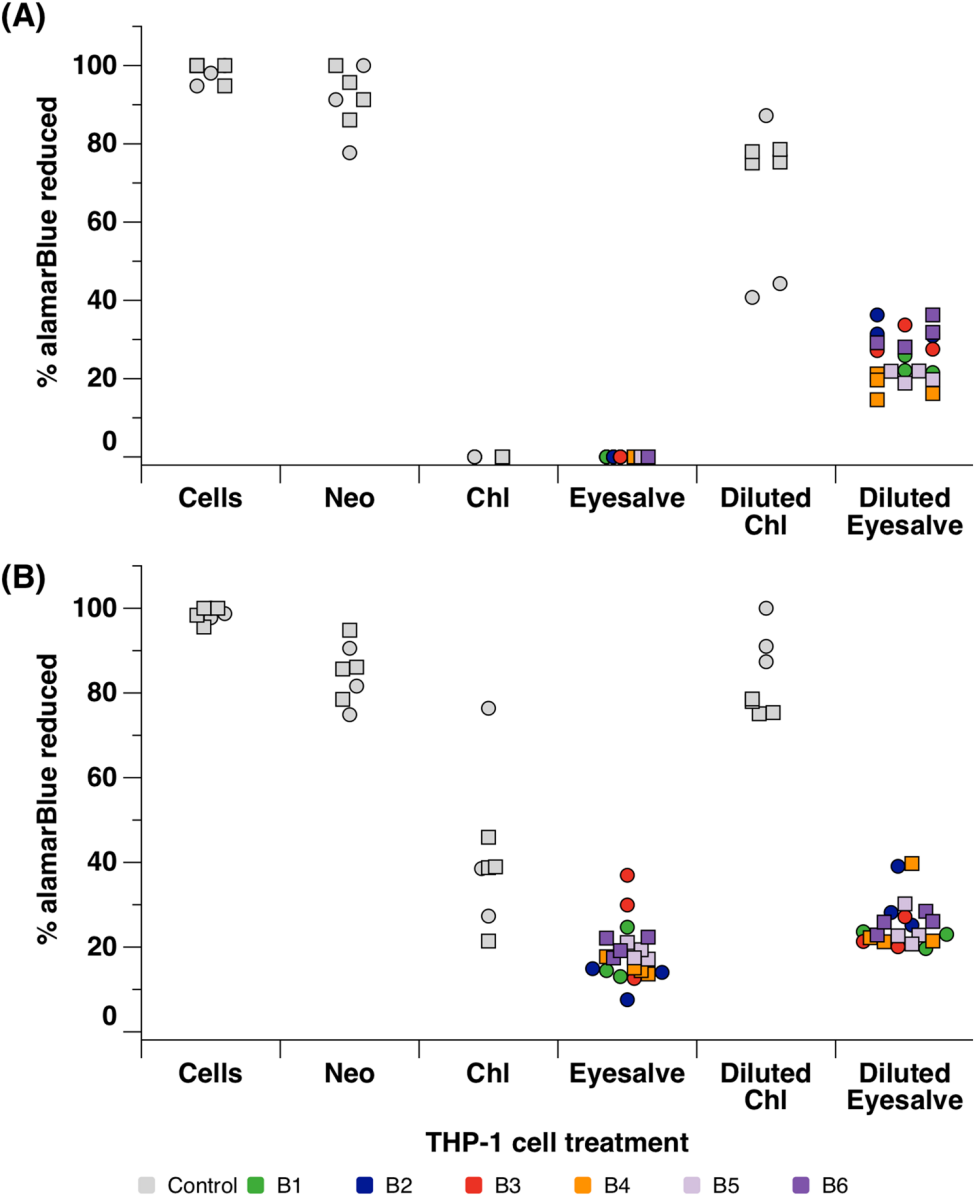
AlamarBlue assay to determine the viability of cells treated with eyesalve. (**A**) HaCaT cells and (**B**) THP-1 cells were treated with six batches of undiluted and 1/10 dilution of the eyesalve (B1–B6) for 24 h; the controls were cells only (untreated), Neosporin (Neo), a safe antibiotic for wound infections and Optrex chloramphenicol (chl) treated cells. The experiment was conducted twice on fresh cultures of cells, with n = 3 or 4 replicates per treatment. Squares and circles denote the two biological repeats, and the colour of eyesalve symbols denotes the batch tested. Note that all replicate HaCaT cultures treated with undiluted chloramphenicol or eyesalve showed no reduction of alamarBlue, thus not all symbols are visible. For HaCaT cells, Neosporin did not affect alamarBlue reduction relative to untreated cells, but alamarBlue reduction was lowered when cells were treated with diluted eyedrops (ANOVA on control treatments only, eliminating experiment: treatment F_2,15_ = 17.5, *p* < 0.001; Dunnett’s test vs. cells only: Neosporin *p* = 0.445, diluted eyedrops *p* = 0.001). All batches of diluted eyesalve caused a greater drop in alamarBlue reduction than diluted eyedrops (ANOVA on diluted treatments only, eliminating experiment: treatment F_6,20_ = 24.8, *p* < 0.001; Dunnett’s test vs. diluted eyedrops: all *p* ≤ 0.012). For THP-1 cells, both Neosporin and chloramphenicol eyedrops reduced alamarBlue reduction relative to untreated cells, but diluted eyedrops did not significantly affect alamarBlue reduction (ANOVA on control treatments only, eliminating experiment: treatment F_3,20_ = 53.6, *p* < 0.001; Dunnett’s test vs. cells only: Neosporin *p* = 0.016, eyedrops *p* < 0.001, diluted eyedrops *p* = 0.517). When batches of undiluted eyesalve were compared with undiluted eyedrops, there was heterogeneity between the six batches (ANOVA on undiluted treatments only, eliminating experiment: treatment F_6,20_ = 6.68, *p* < 0.001; Dunnett’s test vs. eyedrops: B1, B2, B4 *p* ≤ 0.011, B3, B5, B6 p ≥ 0.071). All batches of diluted eyesalve caused a greater reduction in viability than diluted eyedrops (ANOVA on diluted treatments only, eliminating experiment: treatment F_6,20_ = 46.4, *p* < 0.001; Dunnett’s test vs. diluted eyedrops: all *p* < 0.001).

The lack of toxicity of any natural product is a key requirement for applications in humans, so we first determined the toxicity when applied to human cells. Two cell lines, HaCaT and THP-1, were treated with Bald’s eyesalve, and alamarBlue was used to assess the viability of the cells. Viable cells convert the oxidised form of alamarBlue into the reduced form with a change in colour from blue to pink, which is then measured to determine the percentage of cells which are viable (% alamarBlue reduced). As a control, we also treated cells with Neosporin or Optrex chloramphenicol eye drops. Both of these preparations are available without prescription in the USA and the UK. While safe for topical use, chloramphenicol is known to cause death by apoptosis when applied to cell cultures^23^. For the HaCaT cells, treatment with the undiluted eyesalve led to the death of the cells with a similar result in the chloramphenicol control treated cells (Fig. 2a). More viable HaCaT cells were observed in the 1/10 dilution of the eyesalve compared to the undiluted eyesalve. However, the diluted chloramphenicol control had significantly higher viable cells than the diluted eyesalve (ANOVA F_6,20_ = 24.8, *p* < 0.001; Dunnett’s test vs. diluted eyedrops: all *p* ≤ 0.012), Fig. 2a). Whereas, the viability of THP-1 cells treated with both the undiluted and diluted eyesalve were similar (Fig. 2b), but again significantly more viable cells were also observed in the chloramphenicol treated controls (ANOVA on undiluted treatments F_6,20_ = 6.68, *p* < 0.001; Dunnett’s test vs. eyedrops gave varying *p* values depending on batch; ANOVA on diluted treatments F_6,20_ = 46.4, *p* < 0.001; Dunnett’s test vs. diluted eyedrops: all *p* < 0.001).

**Figure 3:**
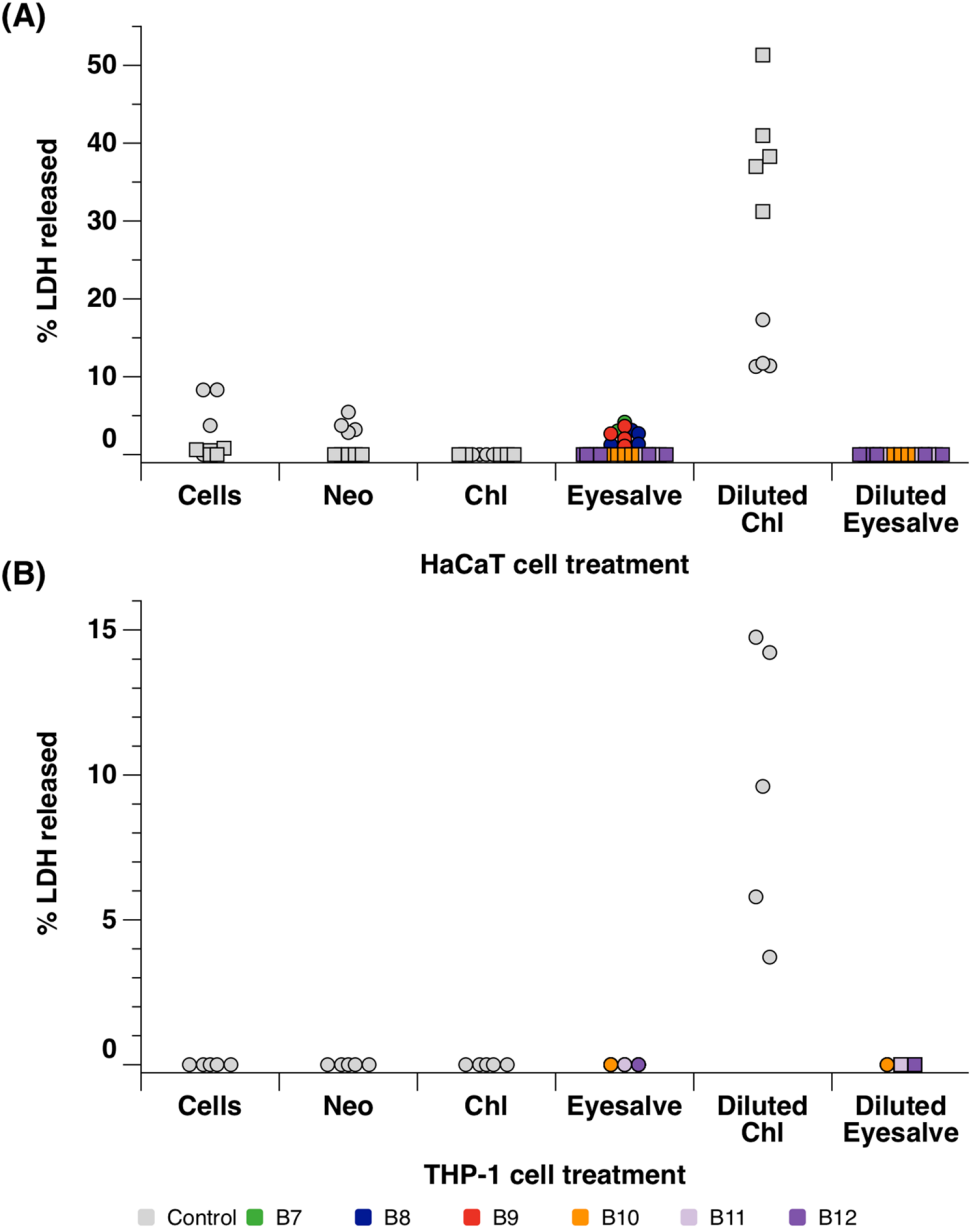
Lactate dehydrogenase assay to determine the cytotoxicity profile of the eyesalve. (**A**) HaCaT cells were treated with six batches, and (**B**) THP-1 cells were treated with three batches, of undiluted and 1/10 dilution of the eyesalve (B7–B12) for 24 h; the controls were cells only (untreated), Neosporin (Neo), a safe antibiotic for wound infections and Optrex chloramphenicol (chl) treated cells. The experiment was conducted twice on fresh cultures of cells, with n = 4 or 5 replicates per treatment. Squares and circles denote the two biological repeats, and the colour of eyesalve symbols denotes the batch tested. Note that for some treatments (HaCaT cells treated with undiluted eyedrops or some batches of eyesalve, THP-1 cells with all treatments except diluted eyedrops), all replicate cultures showed no release of LDH, thus not all symbols are visible. For HaCaT cells, Neosporin had comparable LDH release relative to untreated cells, while diluted eyedrops caused a large increase in LDH release (ANOVA on control treatments only, eliminating experiment: treatment F_3,28_ = 239, *p* < 0.001; Dunnett’s test vs. cells only: Neosporin *p* = 0.9, diluted eyedrops *p* < 0.001). ANOVA was then used to compare HaCaT cells treated with the six batches of undiluted eyesalve with the cell only control, and there was no significant difference between treatments (ANOVA eliminating experiment, F_6,28_ = 1.67 , *p* = 0.134). While diluting the eyesalve did not lead to an increase in LDH release by HaCaT cells, diluted eyedrops led to more LDH release (ANOVA eliminating experiment, F_6,28_ = 94.1 , *p* < 0.001; Dunnett’s test vs. diluted eyedrops for all six batches, *p* < 0.001). There was no measurable LDH release from THP-1 cells treated with Neosporin, undiluted eyedrops, undiluted eyesalve or diluted eyesalve; only diluted eyedrops led to any release of LDH.

Next, we measured the levels of lactate dehydrogenase (LDH) released by the cells after treatment with Bald’s eyesalve. LDH is a cytoplasmic enzyme found in cells and is released when the plasma membrane is damaged due to cells undergoing necrosis, apoptosis or other cellular damage^24^. For HaCaT cells, Neosporin had comparable LDH release relative to the untreated cells, while the diluted eyedrops caused a large increase, in LDH release (Fig. 3a, ANOVA on control treatments F_3,28_ = 239, *p* < 0.001; Dunnett’s test vs. cells only: Neosporin *p* = 0.9, diluted eyedrops *p* < 0.001). When we compared HaCaT cells treated with the six batches of undiluted eyesalve with the cell only control, there was no significant difference between treatments (ANOVA F_6,28_ = 1.67 , *p* = 0.134). While diluting the eyesalve did lead to an increase in LDH release by HaCaT cells, diluted eyesalve led to less LDH release than diluted eyedrops (ANOVA F_6,28_ = 94.1 , *p* < 0.001; Dunnett’s test vs. diluted eyedrops for all six batches, *p* < 0.001. We initially treated the THP-1 cells with three batches of eyesalve and did not observe any LDH being released, including the chloramphenicol treated cells (Fig. S1). We then tested an additional three eyesalve batches in both the undiluted and diluted forms (Fig. 3b). There was no measurable LDH release from THP-1 cells treated with Neosporin, undiluted eyedrops, undiluted eyesalve or diluted eyesalve; only diluted eyedrops led to any release of LDH.

These results from the alamarBlue and LDH assays further demonstrate that while the eyesalve clearly causes some damage to cultured human cells, it may have similar levels of cytotoxicity as the chloramphenicol eye drops used for the treatment of conjunctivitis.

### Bald’s eyesalve causes a brief irritation in the bovine corneal opacity & permeability assay

**Figure 4:**
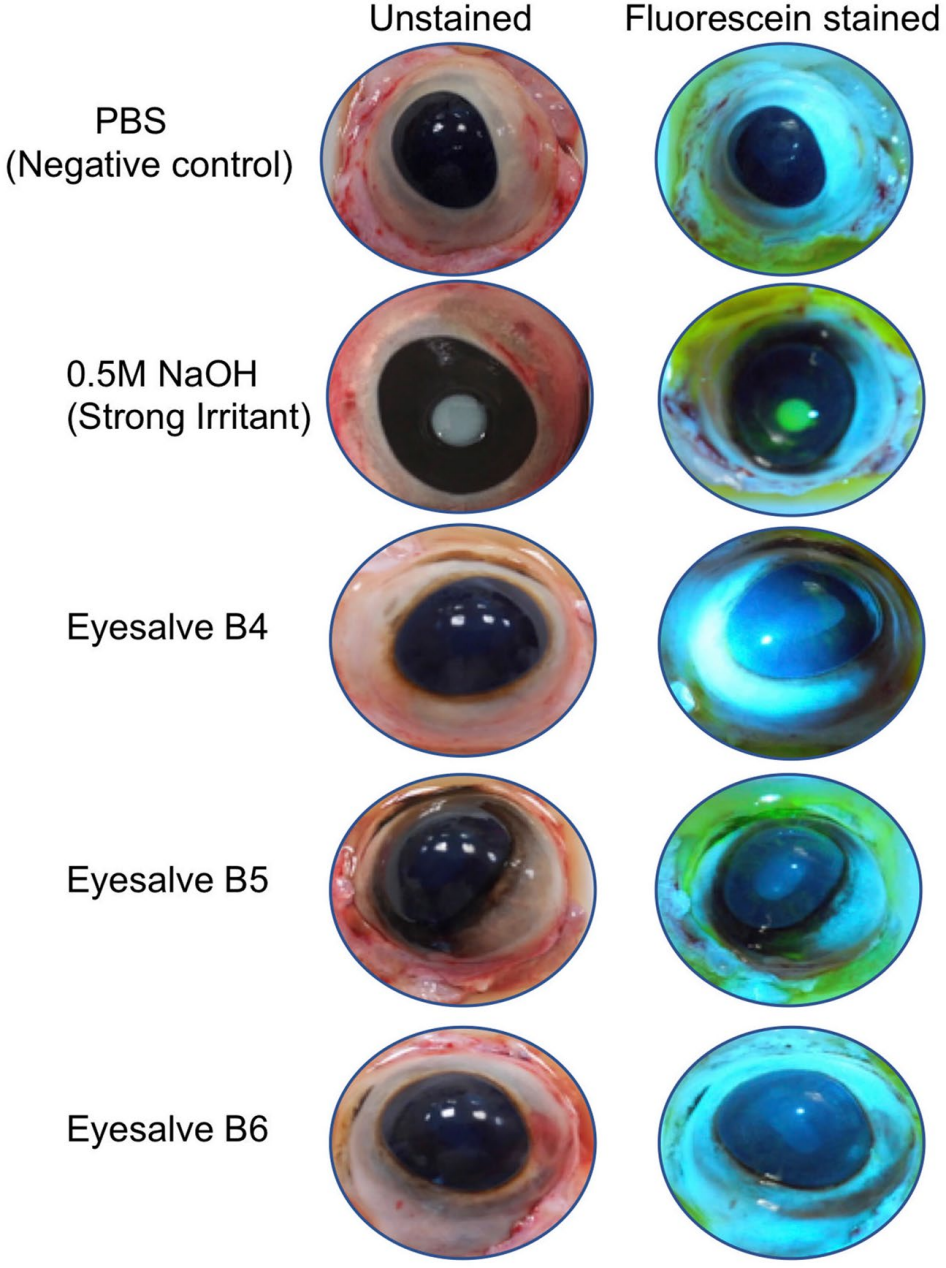
Representative images of bovine eyes treated with different batches of eyesalve. BCOP assay showing unstained and fluorescein stained eye images that were treated with PBS (negative control), 0.5 M NaOH (positive control, strong irritant) and different eyesalve batches (B4, B5 & B6).

Bovine eyes were spotted with the different batches of eyesalve and observed for opacity of the corneal surface followed by washing with PBS. Each eye was then stained with fluorescein and visualized under a cobalt blue filter to reveal any damage and increased permeability caused by the eyesalve. Treatment of the bovine eyes with six batches of eyesalve revealed a transient change to the surface of the eye, which may indicate irritation of the cornea that falls below the criteria for BCOP scoring. The observed change, a slight opacity, resolved within the 10 min incubation of the bovine eye prior to fluorescein staining and was not present at the scoring stage, although opacity and fluorescein staining could be seen in the positive control, (0.5 M NaOH) a strong irritant (Fig. 4). Examination of the corneas for opacity and epithelial damage at the conclusion of the BCOP protocol demonstrated that the six batches of eyesalve caused no irritation (Table 1).

**Table 1:** Bovine corneal opacity and permeability assay cumulative scores for bovine eyes treated with Bald’s eyesalve and the controls. Six batches of eyesalve (B4, B5, B6, B12, B13, B14) were used.

Treatment	Opacity score	Epithelial integrity score	Cumulative score	Description
0.5 M NaOH (positive control)	4	1.5	5.5	Severe
PBS (negative control)	0	0	0	None
B4	0	0	0	None
B5	0	0	0	None
B6	0	0	0	None
B12	0	0	0	None
B13	0	0	0	None
B14	0	0	0	None

### Bald’s eyesalve causes moderate irritation in a slug mucosal irritation assay

To further determine the safety of the eyesalve, the slug mucosal irritation assay was performed with slugs treated with eyesalve, PBS (negative control) or a strong irritant (benzalkonium chloride, positive control). The weights of the slugs, and the weight of mucus secreted after each of the three contacts with the treatments, were compared.

**Figure 5:**
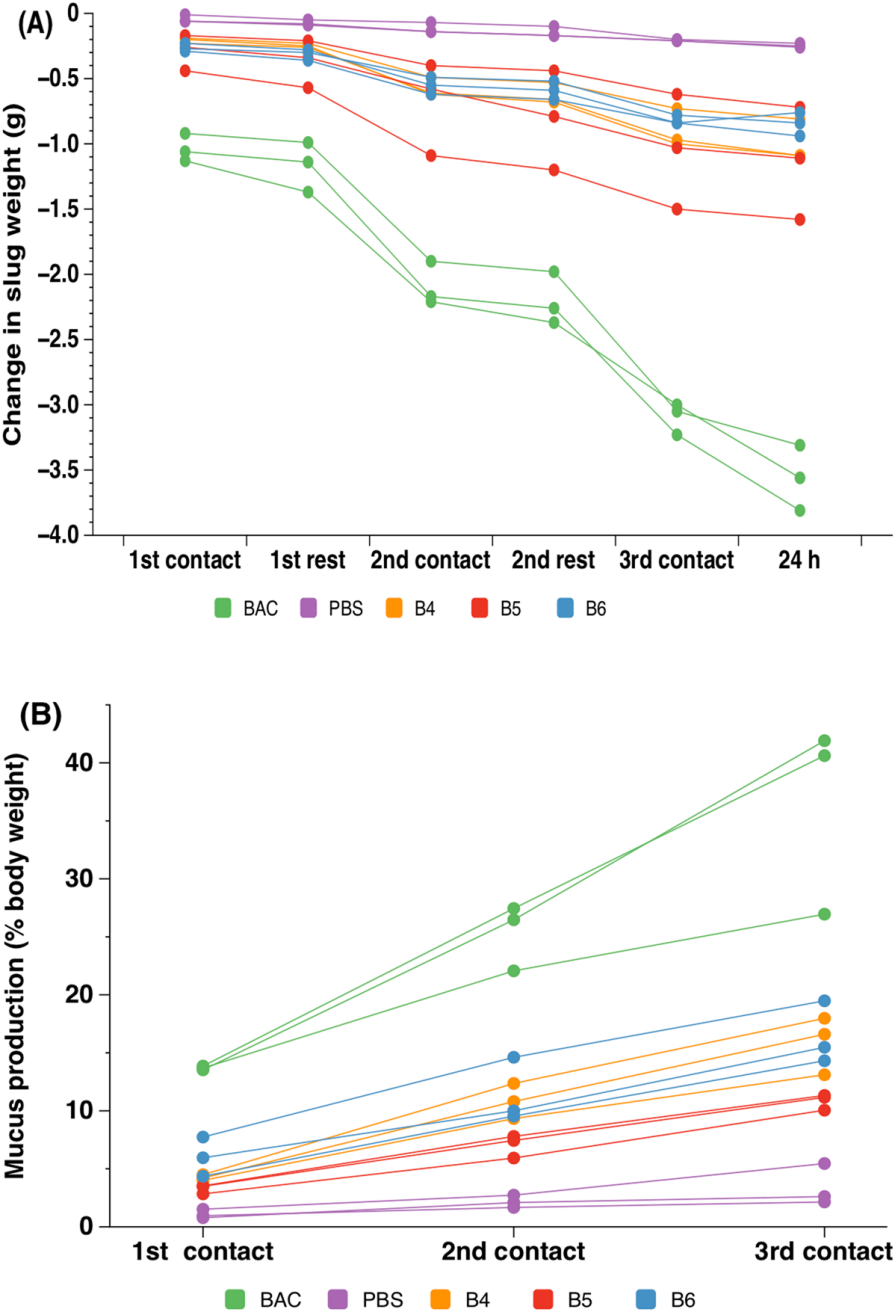
Slug mucosal irritation assay of slugs treated with three batches of eyesalve. (**A**) Slugs used in the irritation assay lost body weight over the course of three consecutive 15 min contacts with the eyesalve (three batches with n = 3 slugs assayed per batch) and a rest of 60 min between the different contacts. The strong irritant positive control, benzalkonium chloride (BAC, n = 3) and non-irritant negative control, PBS (n = 3), were also used. B4, B5, and B6 are the different batches of the eyesalve. More weight loss occurred in the positive control compared to the eyesalve treated slugs (ANOVA on data from 3rd contact point, treatment F_4,10_ = 78.1, *p* < 0.001; Dunnett’s test showed that each eyesalve batch led to less weight loss than the BAC treatment (all *p* < 0.001). (**B**) The cumulative production of mucus, expressed as percentage of each slug’s body weight.. The total cumulative mucus production over the three contact points in the assay is used as a measure of irritant potential when applied to a human eye. A significantly greater amount of mucus was released in the positive control compared to the eyesalve treated slugs (ANOVA on data from 3rd contact point, treatment F_4,10_ = 36.5, p < 0.001; Dunnett’s test showed that each eyesalve batch led to less mucus release than the BAC treatment (*p* < 0.001).

The slugs treated with the positive control lost more weight compared to the eyesalve treated slugs (Fig. 5a, ANOVA on change in slug weight after third contact point, F_4,10_ = 78.1, *p* < 0.001). The positive control, benzalkonium chloride, caused severe irritation to the slugs by the third contact period (as total mucus loss was > 15% body weight) and differed from the eyesalve which caused moderate to severe irritation after the third contact period (total mucus loss 11–21% body weight) (Fig. 5b, ANOVA, F_4,10_ = 36.5, *p* < 0.001). Next, the protein concentration of the mucus secreted after the third contact period was quantified using the NanoOrange protein kit. This showed significantly higher amounts of protein in the positive control compared to the eyesalve treated slugs (ANOVA, F_4,30_ = 15.72, *p* < 0.002, Fig. S2).

### Eyesalve does not interfere with normal wound healing in experimentally wounded mice

**Figure 6:**
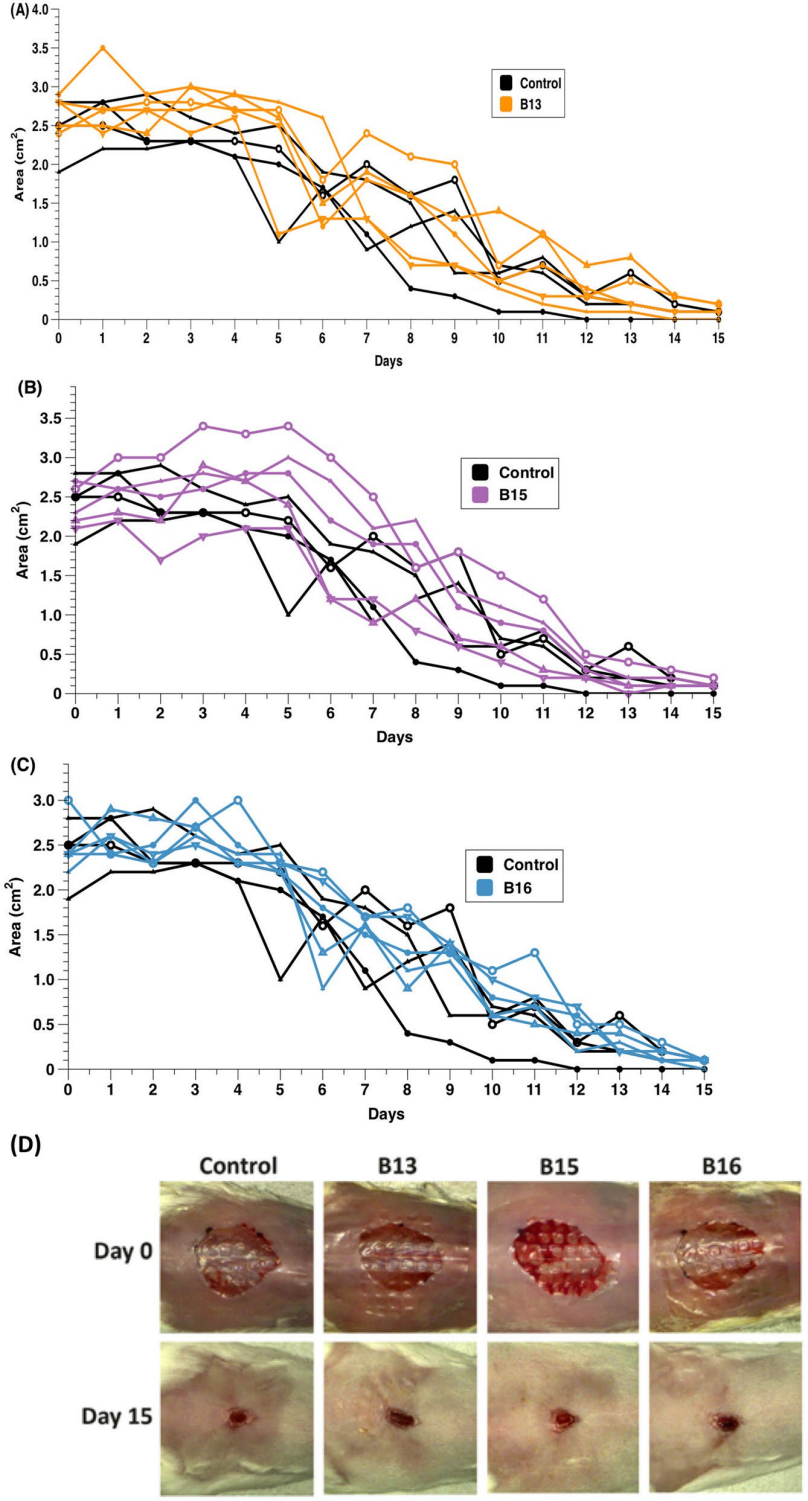
Mice wound closure experiment to determine the safety of Bald’s eyesalve in an in vivo model. (**A**) Area of individual mice wounds treated with 100 µl of three batches of eyesalve (B13, B15 and B16) daily for 15 days. For ease of visual comparison, the mice treated with different batches of eyesalve are shown on separate panels, with three control (sterile water) treated mice superimposed on every panel. There was no difference in wound size between treatment groups at day zero (ANOVA, F_3,15_ = 1.03, *p* = 0.41) or at day 15 (ANOVA, F_3,15_ = 0.825, *p* = 0.5). Linear mixed models were used to compare the dynamics of wound healing among the four treatments, including mouse identity as a random factor. The intercepts of the fitted relationships did not vary between treatments, i.e. wounds were initially of similar sizes in the four groups (*X*^2^ = 2.56, *p* = 0.465, d.f. = 3); there was a significant effect of time on wound area, i.e. wounds healed over time (*X*^2^ = 592, *p* < 0.001, d.f. = 1); and there was no significant difference in healing rates between treatments (time*treatment interaction *X*^2^ = 3.012, *p* = 0.390, d.f. = 3). (**B**) Representative images of the mouse wounds at day 0 and day 15 after treatment with three batches of eyesalve showing a closure of the wounds.

We observed only low levels of cytotoxicity and irritation in cell culture and BCOP assays. The slug mucosal irritation assay suggested that the eyesalve could produce stinging, itching or burning sensations if applied to the eyes. We concluded that an assessment of the safety of the eyesalve in a live vertebrate was justified but using a wound rather than eye or other mucosal model. We previously reported an approximately tenfold drop in viable methicillin-resistant *S. aureus* bacteria in tissue biopsies taken from chronic mouse wound infections treated for 4 h with the eyesalve^10^. In this previous study, we did not treat live mice with the eyesalve or assess any tissue effects, so we aimed to investigate whether the eyesalve would interrupt normal wound healing. We thus used 19 mice, administered a full-thickness surgical excision wound^25^ and treated each mouse by applying 100 µl eyesalve or 100 µl sterile water to the wound. The area of the wound size reduced daily by the different treatments is shown in Fig. 6a–c. There was no significant effect of treatment group on wound size at day zero, as wounds were not consistently bigger or smaller in mice assigned to the different treatments (ANOVA, F_3,15_ = 1.03, *p* = 0.41). All wounds were healed to comparable sizes by day 15 irrespective of treatments (ANOVA, F_3,15_ = 0.825, *p* = 0.5). There were no visible signs of irritation or inflammation, thus no reddening of tissue and the mice did not display signs of irritation.

Example of photographs of wounds treated with the eyesalve at days 0 and 15 are shown in Fig. 6d. More images of the individual mice treated with eyesalve and the control at different days are presented in Fig. S3. We used linear mixed models to compare the rate of wound healing among the four treatments, including mouse identity as a random factor to fit individual relationships between wound area and time for each mouse. There was no difference in healing rates between treatments (testing) for an effect of including a time*treatment interaction in the model to allow the slope of the area on time to vary between treatments (*X*^2^ = 3.012, *p* = 0.39, df = 3). Consistent with the analysis of day 0 data, the intercepts of the fitted relationships did not vary between treatments (*X*^2^ = 2.56, *p* = 0.47, df = 3). However, there was a significant effect of time on wound area, i.e. wounds heal over time (*X*^2^ = 592, *p* < 0.001, df = 1).

## Discussion

Plant-based remedies have been used in ancient times before the advent of traditional antibiotics and could serve as alternatives in the search for new drugs^6,9^. However, it is necessary that any potential product to be used for the treatment of humans is tested for safety and efficacy.

Previously, we have shown that a medieval remedy, Bald’s eyesalve, possesses antibacterial activity against *S. aureus* in both planktonic cultures, biofilms, and in chronic mouse wounds infected with a methicillin-resistant *S. aureus* strain^10^. Recently, we have extended this research by demonstrating the bioactivity of the eyesalve against a range of Gram-positive and Gram-negative organisms in planktonic cultures and biofilms^12^.

Here, we show that Bald’s eyesalve is also effective against a multidrug-resistant *N. gonorrhoeae* strain. *N. gonorrhoeae* is a common cause of ophthalmia neonatorum or neonatal conjunctivitis acquired during delivery from an infected mother. If left untreated, it can lead to corneal perforation and blindness, with neonatal conjunctivitis being a major cause of childhood corneal blindness in developing countries^26–28^. With the increasing occurrence of multidrug-resistant strains of bacteria, an alternative treatment for *N. gonorrhoeae* is very valuable and these experiments show that Bald’s eyesalve has the potential to be developed into a viable alternative.

As the safety profile of the eyesalve has not previously been reported, we used a range of models to assess the irritation potential of the eyesalve with the aim of reducing the number of animals used. First, we performed cytotoxic profiling for cell viability with alamarBlue using HaCaT and THP-1 cells. The undiluted form of the eyesalve was toxic to the HaCaT cells, as was one of the positive controls, Optrex chloramphenicol eyedrops. This suggests that while the eyesalve can cause damage in the cell lines used, it may not cause significant damage to intact eyes since the eyedrops (chloramphenicol, 5 mg/mL) caused a similar level of cell death in the skin cells and are accepted for use to treat eye infections. Similar results have been shown in other studies of undiluted plant-based remedies^29,30^. Low numbers of viable cells were observed in THP-1 cells treated with both the undiluted and the 1/10 dilution of Bald’s eyesalve when compared to the chloramphenicol treated controls.

Levels of lactate dehydrogenase, a cytosolic enzyme released when the plasma membrane is damaged, were low in the treated HaCaT and THP-1 cells. However, the levels were much higher in the 1/10 chloramphenicol treated cells than in the 1/10 eyesalve treated cells. It is pertinent to note that cell lines are in vitro tools for preliminary screening and do not have the complexity of human tissue^31^. Therefore, the results of the alamarBlue and LDH assays demonstrate that while the eyesalve causes some damage to cell lines, this may occur at a sufficiently low level to be insignificant in vivo.

We thus employed whole-organ and whole-animal models that are used in the consumer healthcare industry to assess the irritation potential of drugs and cosmetic products. The BCOP test, an industry-standard alternative to the in vivo Draize rabbit test, is an ex vivo assay that has been used to determine the safety potential of chemicals causing serious eye damage^17,18^. In this assay, the eyesalve did not cause irritation based on the scoring criteria, although some transient mild opacity was observed during the assay, which resolved quickly. However, the slug mucosal irritation assay demonstrated that the eyesalve was irritating to slugs, which have been shown to closely mimic irritation of the human mucosa (Fig. 5a), suggesting that the eyesalve may act as an irritant if applied to the eyes. Further work on the chemical composition of the eyesalve could resolve the conflicting results from the two models and suggest whether any irritation is sufficient to offset any antibacterial benefits, or could be reduced by refinement of the natural product cocktail and/or the use of adjuvants to reduce irritation. If the eyesalve, or a derivative of it, could be safely applied to the eyes this could be particularly relevant for the therapy of neonatal conjunctivitis caused by *N. gonorrhoeae* infections – a condition with a more serious health and economic burden than the styes for which the eyesalve was originally prescribed.

Since the results from the in vitro and ex vivo assays were promising, and because non-mucosal infections sites are likely less susceptible to irritation, we then tested the safety of the eyesalve in a mouse wound model and showed there was no inhibition of wound closure or signs of irritation. This is important as wound healing poses a serious problem, especially in individuals with chronic non-healing diabetic foot infections^32^. As such, being able to design alternative therapies with antimicrobial properties with no effect on wound healing/irritation would be advantageous. This result increases the confidence with which we can suggest Bald’s eyesalve as a candidate for in vivo testing of antibacterial potential.

We used all four ingredients in the preparation of Bald’s eyesalve for the safety testing due to our previous^10^ and recent study^12^ that demonstrated that these ingredients were necessary to achieve the anti-biofilm activity. While it has been shown that an active ingredient, allicin, from garlic has antibacterial activity in planktonic cultures^11,12^, we know from our recent study that other ingredients in addition to allicin are necessary for efficacy against biofilm associated pathogens^12^. Determining and safety testing of other active ingredients necessary for anti-biofilm activity is beyond the scope of this paper. Therefore, future work should focus on this as the results of such research would be a valuable addition to the field of natural product discovery.

With the increase in antimicrobial resistance, alternatives to antibiotics are urgently needed and Bald’s eyesalve could potentially be used to treat bacterial infections through topical application. This would ideally involve the formulation of a defined natural product cocktail produced under good manufacturing processes to ensure reproducible efficacy and safety and to minimise side effects. Key candidates for future testing could include diabetic foot ulcers with a treatment cost average of £7800 per infection annually in the UK^33^. Other potential treatment target include neonatal conjunctivitis, a major cause of childhood corneal blindness in developing countries^26^. Our results are very promising, and the next steps would be to perform rigorous topical testing on healthy humans to determine any potential irritation or toxicity.

## Materials and methods

### Preparation of Bald’s eyesalve

Bald’s eyesalve was prepared using a standard method as previously reported with garlic, onions, bovine bile and white wine^12^. Garlic and brown onions were purchased from supermarkets or greengrocers and is possible that same or different varieties were used. Briefly, the garlic and onions were chopped, pounded together in mortar with a pestle and combined with equal volumes of wine (Pennard’s organic dry white, 11% ABV, Avalon Vineyard, Shepton Mallet) and bovine bile salts (Sigma Aldrich). The bovine bile salts were made up to 89 mg/mL in water and sterilised by exposing to UV radiation for ten minutes. The mixture was stored in the dark at 4 °C for 9 days, after which it was strained, centrifuged and stored in sterilised glass bottles in the dark at 4 °C till when used. The average weight used was 14.1 ± 1.5 g of brown onion and 15.0 ± 1.3 g of garlic per 100 ml of Bald’s eyesalve. Several batches of the eyesalve, made on different days, were used for the experiments.

### Media preparation

Gonococcal (GC) broth was prepared by dissolving 3.75 g of protease peptone (Sigma-Aldrich), 0.25 g of potassium phosphate monobasic (Sigma-Aldrich), 1 g of potassium phosphate dibasic (Sigma-Aldrich) and 1.25 g of sodium chloride (Sigma-Aldrich) into 250 mL of distilled water. The GC broth medium was sterilised by autoclaving at 121 °C for 15 min, cooled to 50 °C before adding 250 µl of the iron and 2.5 mL of the glucose supplement aseptically. GC agar was prepared by dissolving 9 g of GC agar base (CM0367, Oxoid) in 250 mL of distilled water, sterilised by autoclaving, and cooled before adding 250 μl of the iron and 2.5 mL of the glucose supplement.

### Disk diffusion assay

*Neisseria gonorrhoeae* NCCP11945 strain was transferred into the GC broth using a sterile loop and adjusted to 10^7^ cells per mL equivalent to 0.5 McFarland standard. 50 µl of the GC broth containing the NCCP11945 strain was transferred to each GC agar plate and was spread evenly using a sterile plastic spreader. For each plate, four sterile 6 mm blank disks were used and 10 µl of each batch of eyesalve was transferred to the blank disk and distilled water was used as a negative control. The plates were incubated in a 5% CO_2_ incubator at 37 °C and the zones of inhibition were measured with a ruler after 24 h and the experiment was performed in triplicate.

### Log reduction assay

The log reduction method was performed according to Bergsson and colleagues^34^ with minor modifications as in Churchward et al.^19^. *N. gonorrhoeae* strain NCCP11945 was inoculated into the GC broth to an optical density of 0.25–0.30 (520 nm), corresponding to approximately 10^7^ cells per mL. The suspended bacteria (13 μl) was added to 10 μl of each eyesalve and mixed for 2 min. Phosphate buffered saline (PBS) was used as the negative control. After 2 min, 10 μl was transferred to 90 μl of GC broth and serial dilutions performed. The dilutions were plated on a GC agar plate and incubated in 5% CO_2_ at 37 °C for 48 h before counting the colonies.

### Cell culture, media and conditions

Human keratinocytes cell line, HaCaT (P15-P19), a gift from Prof. Mahdad Noursadeghi, University College, London and monocyte-like THP-1 cells (P17-P25), from the European Collection of Authenticated Cell Cultures, ECACC (88081201) were used. HaCaT cells were grown in Dulbecco's Modified Eagle Medium (DMEM) supplemented with 10% heat inactivated foetal bovine serum (FBS, Labtech, UK), and 1% penicillin–streptomycin (10,000 units/mL penicillin, 10 mg/mL streptomycin, Sigma-Aldrich, UK). THP-1 cells were grown in Roswell Park Memorial Institute (RPMI) medium supplemented with 10% heat inactivated FBS, 1% penicillin–streptomycin, 2 mM glutamine (Sigma-Aldrich, UK). All cell lines were maintained in 5% CO_2_ in a humidified incubator at 37 °C with a media change every 1 days.

### AlamarBlue cell viability assay

HaCaT cells (100 µl of 2 × 10^5^ cells/mL) and THP-1 cells (100 µl of 1 × 10^6^ cells/mL) were seeded in 96 well tissue culture plate (Nunc, Thermofisher) for 24 h using DMEM and RPMI media respectively supplemented with 10% heat inactivated FBS in 5% CO_2_ incubator at 37 °C. The media was aspirated from the seeded HaCaT cells and 100 µl of each eyesalve, Neosporin antibiotic ointment (neomycin/polymyxin B/bacitracin, 3.5 mg/mL) and Optrex chloramphenicol (5 mg/mL) were added to the respective wells. The cell only control media was replaced with 100 µl of DMEM media supplemented with 10% heat inactivated FBS and a media only control was included with the various treatments and left to incubate for 24 h. For the 1 in 10 dilution, the eyesalve and controls were diluted in DMEM media supplemented with 10% heat inactivated FBS and 100 µl added to each well. As the THP-1 cells are suspension cells, the media (50 µl) was carefully removed with a pipette and replaced with 50 µl of the various treatments. For the diluted treatment, 10 µl was removed and replaced accordingly with the eyesalve and controls. The alamarBlue assay was performed according to the manufacturer’s instructions with 10 µl alamarBlue (Thermofisher, UK) added to all wells. The absorbance readings at 570 nm and 600 nm after 24 h of treatment were performed on Tecan SPARK 10 M. Three to four replicates per treatment were performed for each batch of eyesalve and controls. The resulting readings were subtracted from the media only controls and converted to percent viability using the recommended formula^35^.

### Lactate dehydrogenase cytotoxicity assay

HaCaT cells (100 µl of 1 × 10^5^ cells/mL) and THP-1 cells (100 µl of 2 × 10^5^ cells/mL) were seeded in 96 well tissue culture plate (Nunc, Thermofisher) for 24 h using DMEM and RPMI media respectively supplemented with 5% FBS in 5% CO_2_ incubator at 37 °C. The various treatments were added to the cells using the approach as per the alamarBlue assay, for 24 h. Four to five replicates per treatment were performed for each batch of eyesalve and controls. Cyquant LDH Cytotoxicity Assay Kit (Thermofisher, UK) was used to determine the lactate dehydrogenase released after the treatment following the manufacturer’s instruction. The absorbance was measured at 490 nm and 680 nm, and LDH activity calculated by subtracting the 680 nm value from the 490 nm value. The resulting readings were subtracted from the media only controls before calculating the percent of LDH released indicating cytotoxicity.

### Bovine corneal opacity and permeability (BCOP) assay

Bovine eyes were collected from a slaughterhouse (ABP, Guildford, Surrey, UK) and transported in sterile saline solution. Before the experiment was carried out, the bovine eyes were examined for epithelium detachment, corneal vascularisation and corneal opacity. The test was performed as previously described^36^. Briefly, 100 µl of the eyesalve was added to the bovine eyes and left for 30 s. A strong irritant positive control of 0.5 M sodium hydroxide and a non-irritant negative control of PBS were included in each experiment. Each eye was rinsed with 10 mL of saline solution and incubated for 10 min in the water bath. The cornea was examined under a cobalt blue filter (465–490 nm, peak 480 nm) following sodium fluorescein treatment (2% (w/v), pH 7.4). Each eye was scored using the scoring matrix in Supplementary Table 1^17^.

### Slug mucosal irritation assay

This was performed as detailed by Lenoir and colleagues^21^. Slugs (*Arion* spp.) were collected from a family house garden (in Ham around Richmond riverside, UK). The slugs were fed carrot, lettuce and cucumber purchased from a local supermarket (Sainsbury’s, Penrhyn Road, Kingston upon Thames, UK). Two days before the start of the experiment, the slugs were isolated, weighed and examined for macroscopic injuries. Each slug was placed in individual boxes with paper towels moistened with PBS (pH 7.4). The slugs were kept at 15 °C and the body was wet daily with 30 µl PBS. For the experiment, there are three contact periods of 15 min with the eyesalve followed by 60 min of rest period. Three different batches of eyesalve (100 µl) were used along with a strong irritant positive control of 1% w/v benzalkonium chloride and a non-irritant negative control of PBS. The amount of mucus produced and the weight of the slugs before and after each of the three contact periods were recorded. The mucus produced during each contact period was weighed and the weight converted to percent of the slug’s body weight by dividing the weight of the mucus produced by the initial body weight of the slug. The classification model of Lenoir et al. was used to determine the level of irritation caused by the eyesalve^21^. Test substances resulting in a total (cumulative) mucus production over the three contact periods of ≤ 3% were classified as causing no discomfort. Those between 3 and 8% were classified as mild discomfort, between 8 and 15% was associated with moderate discomfort and severe discomfort for test substances with mucus production > 15%^21^. The protein concentration of the mucus released during the irritation test after the third contact period was analysed using NanoOrange protein kit (Invitrogen) with infinite M200 pro microplate reader with the excitation wavelength of 485 nm and an emission wavelength of 590 nm. The fluorescence results were converted to protein concentrations using a NanoOrange standard curve obtained using bovine serum albumin.

#### Mouse wound closure experiments

This study was carried out in strict accordance with the recommendations in the Guide for the Care and Use of Laboratory Animals of the National Institutes of Health. The protocol was approved by the Institutional Animal Care and Use Committee of Texas Tech University Health Sciences Center (protocol number 07044). Mice wound closure experiments were performed as previously described^25^. Briefly, 19 female Swiss Webster mice were fully anaesthetized with Nembutal (pentobarbital), hair was removed with shaving and Nair (Church & Dwight), and lidocaine was injected subcutaneously at the site of excision prior to manually fully excising approximately 1.5 × 1.5 cm area of all skin layers. The open wound was then covered with an OPSITE bandage (Smith and Nephew, UK). Images of non-infected wounds were taken at day 0 prior to treating the wounds with 100 µl of the different batches of eyesalve or water (control) with a 25G tuberculin syringe under the bandage to coat and remain on top of the open wound. Each day, the dressing was gently removed, and a further 100 µl of eyesalve or water was added. Images were taken with a Silhouette wound imaging and documentation system (Aranz Medical) every 24 h after anaesthetizing mice with isoflurane. This continued for 15 days, by which time all the superficial wounds had healed.

## Statistical analysis

All data were analysed with RStudio version 1.1.447^37^ or R version 3.5.1^38^. Data that fit the assumptions of linear modelling, or which could be transformed to fit the assumptions, were analysed using linear models followed by ANOVA and Dunnett’s test for multiple comparisons against control groups (using the *car* package^39^ when Type II sums of squares were required) or by linear mixed models using the *lme4* package^40^. Data which could not be analysed parametrically (zones of inhibition) were analysed using Kruskal–Wallis tests. Raw data and associated R code will be made available with the accepted version of this manuscript.

## Supplementary information

Supplementary Information.

Supplementary Dataset.

## Data Availability

Raw data and associated R codes will be made available with the accepted version of this manuscript.
