# Supplementary material for: The safety profile of Bald’s eyesalve for the treatment of bacterial infections
Source: Sci Rep. 2020 Oct 15;10:17513. doi: 10.1038/s41598-020-74242-2 (PMC7562736; doi:10.1038/s41598-020-74242-2)
Supplement: Supplementary file 1 — Supplementary Information. [file 41598_2020_74242_MOESM1_ESM.pdf]

The safety profile of Bald's eyesalve for the treatment of bacterial infections

Blessing O. Anonye[1*], Valentine Nweke[2], Jessica Furner-Pardoe[1,7], Rebecca Gabrilska[3], Afshan Rafiq[2], Faith Ukachukwu[2], Julie Bruce[4], Christina Lee[5], Meera Unnikrishnan[6], Kendra P. Rumbaugh[3], Lori A. S. Snyder[2], Freya Harrison[1]

[1] School of Life Sciences, University of Warwick, Coventry, UK

[2] School of Life Sciences, Pharmacy, and Chemistry, Kingston University, Kingston upon Thames, UK

[3] Department of Surgery, Texas Tech University Health Sciences Centre School of Medicine, Texas, USA

[4] Warwick Clinical Trials Unit, Warwick Medical School, University of Warwick, Coventry, UK

[5] School of English and Centre for the Study of the Viking Age, University of Nottingham, Nottingham, UK

[6] Microbiology and Infection Unit, Warwick Medical School, University of Warwick, Coventry, UK

[7] Warwick Medical School, University of Warwick, Coventry, UK

[#]Present address: School of Medicine, University of Central Lancashire, Preston, PR1 2HE

[*] Corresponding author: Blessing Anonye b.anonye@warwick.ac.uk

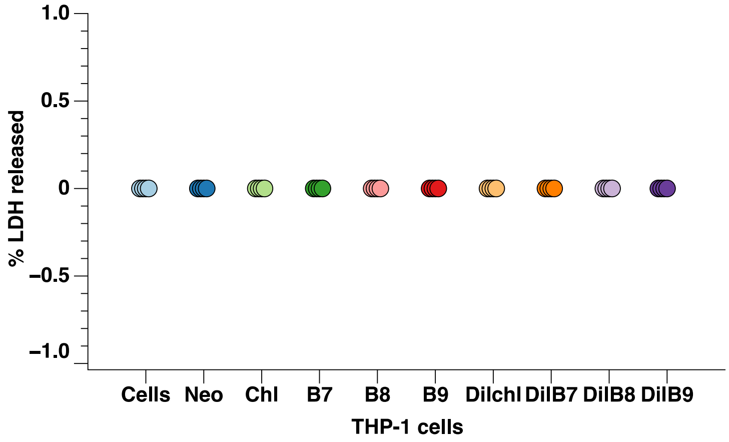

Figure S1: Lactate dehydrogenase assay of THP-1 cells treated with eyesalve. THP-1 cells were treated with three batches of eyesalve (B7, B8 and B9) in the undiluted and diluted (1/10) forms. The controls include cells only (untreated), Neosporin (Neo), a safe antibiotic for wound infections and Optrex^{TM} chloramphenicol (chl) treated cells (n = 4 replicates). The preface “dil” represents cells treated with a 1 in 10 dilution of either the chloramphenicol or the different eyesalve batches.

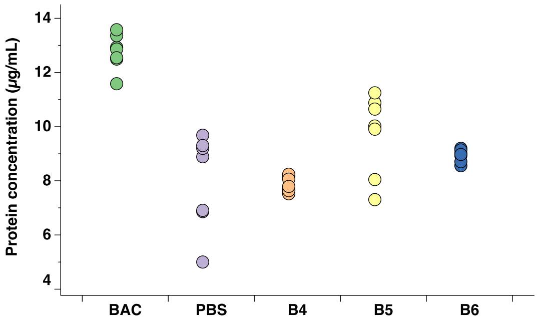

Figure S2: Protein concentration of mucus produced from slugs treated with eyesalve. Slugs were treated with three batches of eyesalve (B4, B5 and B6) and the protein concentration of the mucus measured using the NanoOrange kit. The positive control is benzalkonium chloride (BAC) and the negative control, phosphate buffered saline, PBS. ANOVA found significant higher protein concentration in the

positive control compared to the eyesalve treated slugs followed by Dunnett's test for multiple comparison, $F4,30 = 15.72$, $p < 0.002$, $n = 7$ replicates).

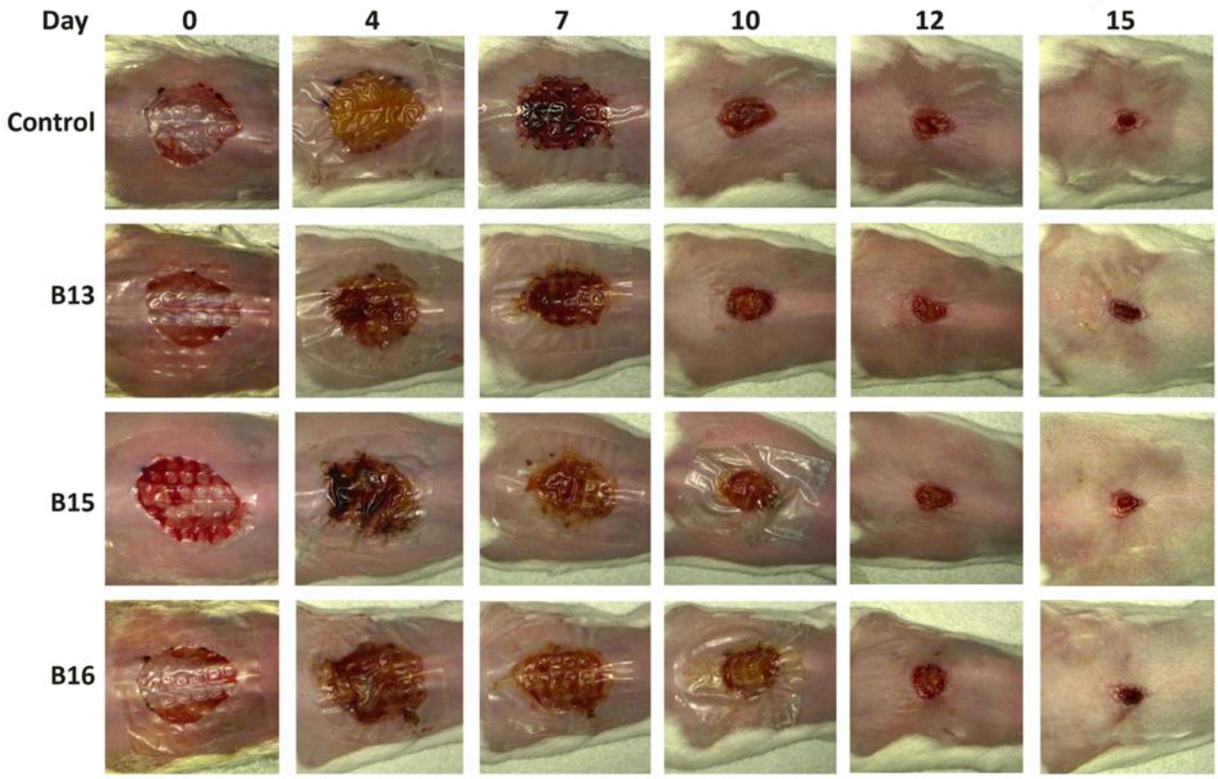

Figure S3: Images of the mouse wounds at different days of treatment with three batches of eyesalve showing closure of the wounds. The control is sterile water.

Supplementary Table 1: Bovine corneal opacity and permeability assay scoring matrix (modified from Van Erp & Weterings, 1990). Opacity is scored visually based on what is seen with the white light/unstained and epithelial integrity is scored following fluorescein staining visualised with a cobalt blue filtered light.

Opacity	Score	Epithelial integrity	Score	Cumulative score	Description
**None**	0	None	0	≤ 0.5	None
**Slight**	1	Diffuse and weak	0.5	0.6 - 1.9	Slight
**Marked**	2	Confluent and weak	1	2.0 - 4.0	Moderate
**Severe**	3	Confluent and intense	1.5	> 4	Severe
